# Supplementary material for: Genome-resolved metagenomics reveals site-specific diversity of episymbiotic CPR bacteria and DPANN archaea in groundwater ecosystems
Source: Nat Microbiol. 2021 Jan 25;6(3):354–65. doi: 10.1038/s41564-020-00840-5 (PMC7906910; doi:10.1038/s41564-020-00840-5)
Supplement: Supplementary file 1 — Reporting Summary [file 41564_2020_840_MOESM1_ESM.pdf]

nature research

Corresponding author(s): Jillian Banfield

Last updated by author(s): Nov 17, 2020

Reporting Summary

Nature Research wishes to improve the reproducibility of the work that we publish. This form provides structure for consistency and transparency in reporting. For further information on Nature Research policies, see our Editorial Policies and the Editorial Policy Checklist.

Statistics

For all statistical analyses, confirm that the following items are present in the figure legend, table legend, main text, or Methods section.

n/a	Confirmed	
☐	☒	The exact sample size (*n*) for each experimental group/condition, given as a discrete number and unit of measurement
☐	☒	A statement on whether measurements were taken from distinct samples or whether the same sample was measured repeatedly
☐	☒	The statistical test(s) used AND whether they are one- or two-sided<br>*Only common tests should be described solely by name; describe more complex techniques in the Methods section.*
☒	☐	A description of all covariates tested
☐	☒	A description of any assumptions or corrections, such as tests of normality and adjustment for multiple comparisons
☐	☒	A full description of the statistical parameters including central tendency (e.g. means) or other basic estimates (e.g. regression coefficient) AND variation (e.g. standard deviation) or associated estimates of uncertainty (e.g. confidence intervals)
☐	☒	For null hypothesis testing, the test statistic (e.g. *F*, *t*, *r*) with confidence intervals, effect sizes, degrees of freedom and *P* value noted<br>*Give P values as exact values whenever suitable.*
☒	☐	For Bayesian analysis, information on the choice of priors and Markov chain Monte Carlo settings
☒	☐	For hierarchical and complex designs, identification of the appropriate level for tests and full reporting of outcomes
☒	☐	Estimates of effect sizes (e.g. Cohen's *d*, Pearson's *r*), indicating how they were calculated

Our web collection on statistics for biologists contains articles on many of the points above.

Software and code

Policy information about availability of computer code

Data collection	Gatan Microscopy Suite (v 3.4.1), SerialEM (v 3.7)
Data analysis	BBTools (v 38.78); Sickle (v 1.33); MEGAHIT (v 1.2.9); IDBA-UD (v 1.1.3); bowtie2 (v 2.3.5.1); Prodigal (v 2.6.3); usearch (v 10.0.240); 16SfromHMM.py (available at https://github.com/christophertbrown/bioscripts); tRNAscan-SE (v 1.3.1); CONCOCT (v 1.1.0); Maxbin2 (v 2.2.7); Abawaca (v 1.07); DASTool (v 1.1.1); ggkbase (https://ggkbase.berkeley.edu/); dRep (v 2.5.3); METABOLIC (v 1.3); IMOD (v 4.9); ImageJ (v 2.0.0); iRep (v 1.1.14);

For manuscripts utilizing custom algorithms or software that are central to the research but not yet described in published literature, software must be made available to editors and reviewers. We strongly encourage code deposition in a community repository (e.g. GitHub). See the Nature Research guidelines for submitting code & software for further information.

Data

Policy information about availability of data

All manuscripts must include a data availability statement. This statement should provide the following information, where applicable:

- Accession codes, unique identifiers, or web links for publicly available datasets
- A list of figures that have associated raw data
- A description of any restrictions on data availability

SRA accession numbers for metagenome reads are in SI Table 18. All metagenome-assembled genomes from this study are deposited in NCBI under Bioproject PRJNA640378. The genomes are also available at: http://ggkbase.berkeley.edu/all_nc_groundwater_genomes (please note that it is necessary to register for an account by provision of an email address prior to download).

Field-specific reporting

Please select the one below that is the best fit for your research. If you are not sure, read the appropriate sections before making your selection.

☐ Life sciences ☐ Behavioural & social sciences ☒ Ecological, evolutionary & environmental sciences

For a reference copy of the document with all sections, see nature.com/documents/nr-reporting-summary-flat.pdf

Ecological, evolutionary & environmental sciences study design

All studies must disclose on these points even when the disclosure is negative.

Study description	This study performs genome-resolved metagenomics analysis and cryo-electron microscopy on bacterial and archaeal communities in 8 groundwater sites.
Research sample	For the metagenomics portion of the study, the research samples are metagenomes sequenced from 8 groundwater sites in northern California. For the cryo-electron microscopy, the sample is groundwater from one of these sites concentrated by tangential flow filtration.
Sampling strategy	At each site, 400-1200 L of groundwater (planktonic portion) was pumped onto filters from which DNA was extracted. For cryo-electron microscopy, 20 L of groundwater was pumped and concentrated to <5 mL using tangential flow filtration.
Data collection	Extracted DNA was sequenced on either HiSeq 4000 or NovaSeq 6000 platforms, at either the California Institute for Quantitative Biosciences' (QB3) genomics facility or the Chan Zuckerberg Biohub's sequencing facility.
Timing and spatial scale	Groundwater sampling dates by site: Ag (03/17, 09/17, 11/17, 02/18, 06/18), Pr1 (12/18), Pr2 (05/19), Pr3 (11/17, 09/18), Pr4 (09/18), Pr5 (05/19), Pr6 (05/19), Pr7 (11/18).
Data exclusions	No data were excluded from analysis.
Reproducibility	No explicit measures were taken to ensure reproducibility of assembled genomes from each site. Time series sampling of Ag groundwater show that similar genomes are recovered from each time point.
Randomization	Genomes were taxonomically classified based on a phylogenetic tree of concatenated ribosomal proteins, allowing us to categorize genomes as CPR bacteria, non-CPR bacteria, DPANN archaea, and non-DPANN archaea.
Blinding	Blinding was not relevant to our study.

Did the study involve field work? ☒ Yes ☐ No

Field work, collection and transport

Field conditions	All groundwater was pumped from shallow wells (<100 m deep).
Location	Sites Pr1 through Pr7 are located in Lake/Napa County, California, while site Ag is located in Modesto, California.
Access & import/export	Private sites were sampled with explicit permission from the property owner.
Disturbance	To our knowledge, our groundwater sampling did not cause any disturbance.

Reporting for specific materials, systems and methods

We require information from authors about some types of materials, experimental systems and methods used in many studies. Here, indicate whether each material, system or method listed is relevant to your study. If you are not sure if a list item applies to your research, read the appropriate section before selecting a response.

Materials & experimental systems

n/a	Involved in the study
☒	☐ Antibodies
☒	☐ Eukaryotic cell lines
☒	☐ Palaeontology and archaeology
☒	☐ Animals and other organisms
☒	☐ Human research participants
☒	☐ Clinical data
☒	☐ Dual use research of concern

Methods

n/a	Involved in the study
☒	☐ ChIP-seq
☒	☐ Flow cytometry
☒	☐ MRI-based neuroimaging
